# Acoustic Characterization of Edzna: A Measurement Dataset

**DOI:** 10.1038/s41597-023-02577-2

**Published:** 2023-09-28

**Authors:** Gustavo Navas-Reascos, Guillermo Wilhelm-deAlba, Luz María Alonso-Valerdi, David I. Ibarra-Zarate

**Affiliations:** 1 Tecnologico de Monterrey, Escuela de Ingeniería y Ciencias, Ave. Eugenio Garza Sada 2501, Monterrey, N.L. 64849 Mexico; 2 Universidad Nacional Autónoma de México, Centro de Estudios Mayas, Proyecto Universos Sonoros Mayas, Av. Universidad 3000, Ciudad Universitaria, Coyoacán, CDMX, 04510 Mexico City, Mexico

**Keywords:** Engineering, Scientific community

## Abstract

Acoustic characterizations of different locations are necessary to obtain relevant information on their behavior, particularly in the case of places that have not been fully understood or which purpose is still unknown since they are from cultures that no longer exist. Acoustic measurements were conducted in the archaeological zone of Edzna to obtain useful information to better understand the customs and practices of its past inhabitants. The information obtained from these acoustic measurements is presented in a dataset, which includes measurements taken at 32 points around the entire archaeological zone, with special attention given to the Main Plaza, the Great Acropolis, and the Little Acropolis. Two recording systems were used for this purpose: a microphone and a binaural head. As a result, a measurement database with the following characteristics was obtained: it comprises a total of 32 measurement points with 4 different sound source positions. In total, there are 297 files divided into separate folders. The sampling frequency used was 96 kHz, and the files are in mat format.

## Background & Summary

This dataset refers to the acoustic measurements of the archaeological zone of Edzna in Campeche, Mexico, which were recorded for site characterization purposes. A previous review of the literature^1^ identified twenty-eight studies related to acoustic measurements from various locations around the world between 2016–2022. Twenty of them were conducted in Europe^2–21^, four of them in Asia^22–25^, three of them in America^26–28^, and the remaining one in Africa^29^. This indicates that only three of the studies were conducted in the Americas, and none of them focused on the Mayan culture, despite its significant role in the development of the inhabitants in America. Therefore, this study was undertaken to address this research gap, and provide insight into the acoustic characteristics of the Edzna site. Additionally, it should be noted that there are no previous databases with these characteristics.

**Fig. 1:**
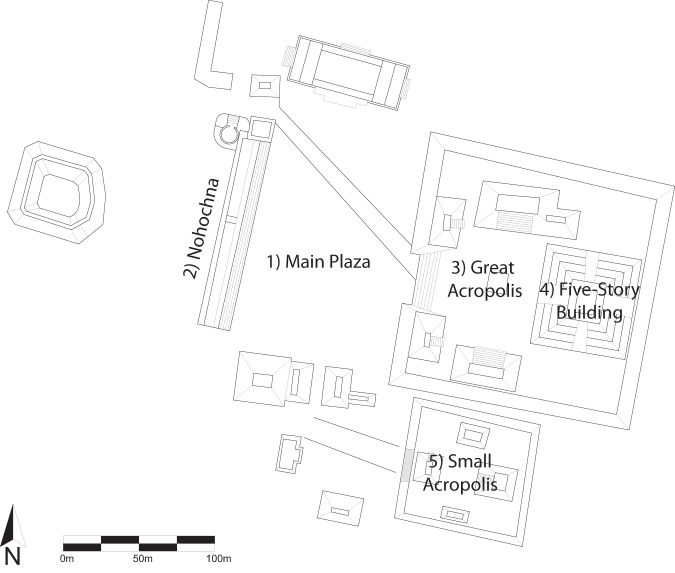
Edzna map and the main places around it. These are: (1) the main plaza, likely used for ceremonies; (2) Nohochna, which probably served as bleachers for seating; (3) the Great Acropolis, the most important and private area of the complex; (4) the Five-Story Building, the most significant pyramid in the entire complex; and (5) the Small Acropolis, a place for resting.

Edzna is an archaeological site belonging to the ancient Mayan civilization, located in the southeastern region of Yucatan Peninsula in the state of Campeche, Mexico. The site is situated approximately 55 kilometers southeast of the city of “San Francisco de Campeche”. Edzna was first discovered in the early 1900s, and since then, it has been an important site for archaeological research and exploration^30^. Its impressive structures, including the Great Acropolis, Nohochna and the Ballcourt, are testament to the rich history and culture of the Mayan people. Some of the main places around the archaeological zone are: (1) Main Plaza, (2) Nohochna, (3) Great Acropolis, (4) The Five-Story building, (5) Small Acropolis. See Fig. 1.

Mexico, a country blessed with a wealth of cultural and historical heritage, boasts an abundance of archaeological sites. Archaeoacoustics-based research has allowed to identify the favored spots for performing ceremonial music in Palenque^31^, and explore the intriguing sound effects within the structures of Chichen Itza^32,33^. Moreover, researchers have discovered remarkable speech intelligibility at Cañada de la Virgen^34^.

Edzna, is a former site of the Mayan culture. The acoustic properties of this architectural complex embody intangible heritage, encompassing a collection of customs, knowledge, traditions, and living expressions transmitted through oral traditions, rituals, festive events, and practices associated with nature and the universe. This exploration grants profound insights into Mayan society, enabling a deeper understanding of the religious ceremonies held, the intricacies of political governance^35^.

The Great Acropolis had several constructions, where various ritual activities took place, such as the Temazcal and the platform located at the center of the courtyard, in addition to the Five-story building. The Nohochna could have had astronomical functions and, at the same time, seemed suitable for conducting various gatherings, such as political or military councils, religious or educational activities, various ceremonies like dances, banquets, or processions, as well as activities related to record-keeping, storage, and distribution of materials or objects^30^. Regarding the archaeological materials that might provide evidence of musical or sound activities in Edzná, there are some indications that can be found in the iconography of the stelae and other iconographic and epigraphic materials from Edzna. For example, stela 13 seems to depict a drum, and stela 18 shows a governor dancing^36^.

**Fig. 2:**
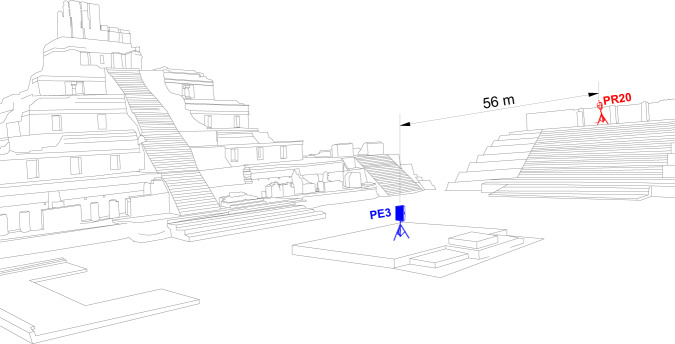
Schematic overview of the measurements carried out. As an example, the emission point PE3 and the recording point PR20 located in the Great Acropolis area are herein depicted. The building in the background corresponds to the Five-Story building.

To carry out the acoustic characterization of Edzna, the equipment to be used had to be calibrated beforehand. This was done prior to visiting the site. Once at the archaeological zone, it was necessary to determine the locations for both: the sound source and, the microphones. Background noise measurements of the site were then taken, following the guidelines specified in the ISO 3382-1:2009 standard^37^. Then the acoustic measurements of the place were performed using two types of signals: (1) Maximum Length Sequence (MLS) and (2) Logarithmic Sine Sweep (LSS) generated by a sound source Behringer DR115DSP. Two different sound capture systems were used for the recordings: (1) a Shure MX 150 microphone, and (2) a binaural head Neumann KU 100. These systems were used to ensure that accurate and reliable measurements were obtained for the acoustic characterization of the site. The Fig. 2 presents the schematic overview of the measurements.

The data herein presented allow to obtain various acoustic parameters of the archaeological zone at different measurement points. This includes parameters such as Early Decay Time (EDT), Reverberation Time (RT_60_), voice Clarity (C_50_), instrument Clarity (C_80_), or Definition (D_50_), which are essential for achieving a comprehensive acoustic characterization of the site.

Additionally, the data collected can be used to auralize different types of sounds, such as musical instruments or sound effects. To have an aural experience where sounds could be listened to as they are perceived in the archaeological zone, without the need of physical present, sound achievable. Such auralizations could help to enhance the experience of the site and provide a better understanding of the acoustic properties of the ancient structures. Moreover, this database can be utilized for machine learning purposes to predict or classify several situations related to the archaeological zone, such as acoustic environment classification, acoustic anomaly detection, human activity recognition, among others.

## Methods

For this study, the materials and equipment used to collect measurements included an HP ProBook 640 G2 laptop, a Behringer UMC 404 HD audio interface, and a Behringer DR115DSP speaker. The microphone employed was the Shure MX 150 B/O, which was equipped with its windscreen. Additionally, a Neumann KU 100 binaural head, a Bruel & Kjaer type 2270 sound level meter with a microphone head 4189 and a preamplifier ZC 0032, along with a Bruel & Kjaer 4231 acoustic calibrator were utilized.

Furthermore, to ensure a continuous power supply, a Baldr Pioneer 330 portable power station was used. Environmental temperature measurements were taken using a Steren TER-100 environmental thermometer and a 50-meter metric measuring wheel. Additionally, Matlab was employed to develop these measurements.

**Fig. 3:**
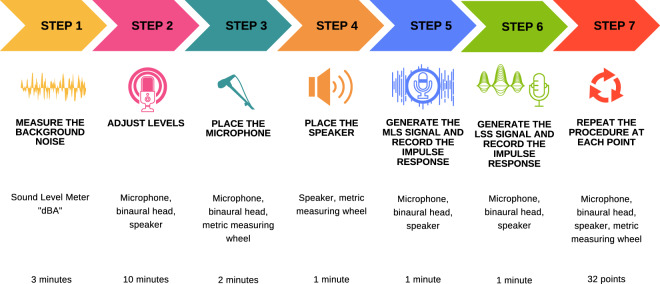
Experimental procedure to record the acoustic measurements of the archaeological site, Edzna (Campeche, Mexico).

The experimental procedure is illustrated in Fig. 3. This one was undertaken as follows. First, background noise measurements were taken, and it was identified that the background noise in the area was 35 dBA, which remained constant throughout the archaeological place. Then, system levels were adjusted, i.e., the speaker output level was checked, and the microphone level was verified to guarantee it was receiving enough level without reaching saturation. A SPL of 115 dB at 1 m was measured to provide enough level for subsequent acoustic measurements.

**Fig. 4:**
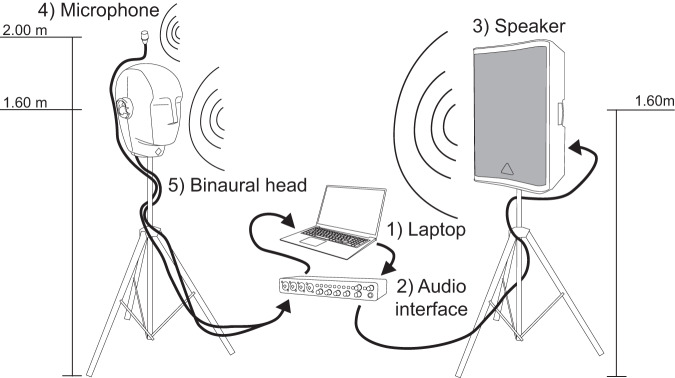
Electroacoustic system configuration. Five items are identified: (1) laptop; (2) audio interface; (3) speaker; (4) microphone; (5) binaural head.

Having adjusted the levels, the microphones were positioned. A total of three were used, two in the binaural head and one located over the head. The binaural head microphones (located inside the ears) were placed at a height of 1.6 m, and the other microphone was placed 40 cm above the head at a height of 2 m. In none case, the microphones were placed less than 1 m from any reflective surfaces (e.g., walls) to comply with ISO 3382-1:2009 standard^37^. Once the microphones were positioned, the speaker was placed at a height of 1.6 m from the center of the larger driver. See Fig. 4. To properly capture all the information of the reflections in the location, the speaker was placed in two positions at each measurement point: 1) at 0° and 2) at 180° from the initial configuration.

**Fig. 5:**
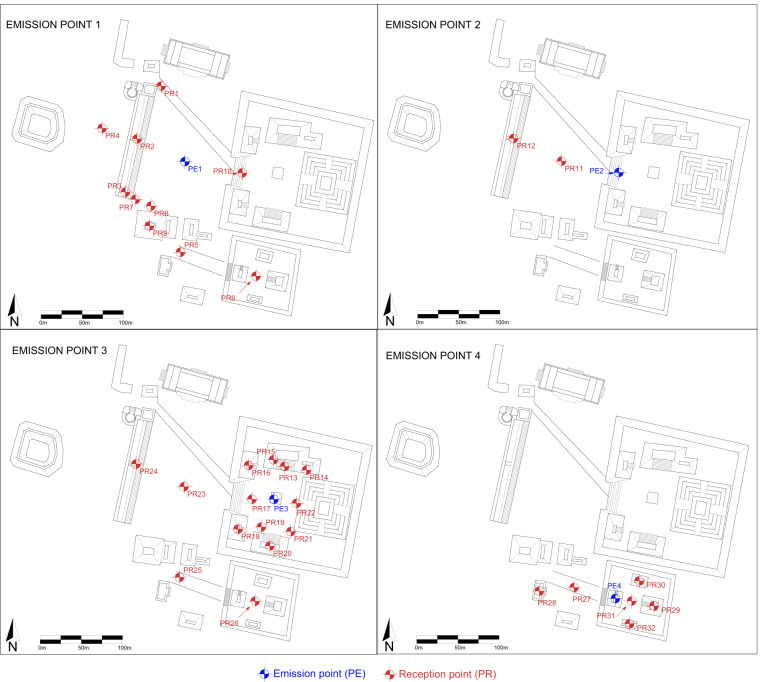
Acoustic measurement points in Edzna. In total, 32 reception and 4 emission points were used.

Once both the microphones and the speaker were in place, measurements were taken by generating a MLS signal and recording the signals. Subsequently, a LSS was generated, and the recordings were repeated. The average weather conditions during the measurements were the following: relative humidity 76%, wind speed 11.5 km/h, and temperature 34 °C. Finally, this procedure was repeated 32 times, one for each measurement point. The measurement points are depicted in Fig. 5.

It is important to note that these measurements were taken in an area with no access to electrical power. Consequently, a Portable Power Station was requited, what limited the measurement options.

## Data Records

**Fig. 6:**
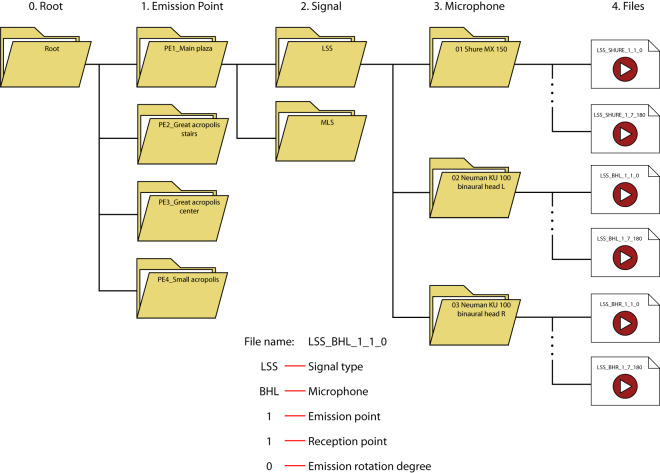
Data organization. The information was organized into four levels: the first one is the root, that contains all the information; the second one organizes the four sound emission points, the third one groups the two types of signals, and finally, the fourth one separates the information by microphone type, among the three options.

The data has been organized maintaining the structure presented in Fig. 6.

The database is available at https://data.mendeley.com/datasets/2vy7f87cdx/1^38^.

## Technical Validation

**Fig. 7:**
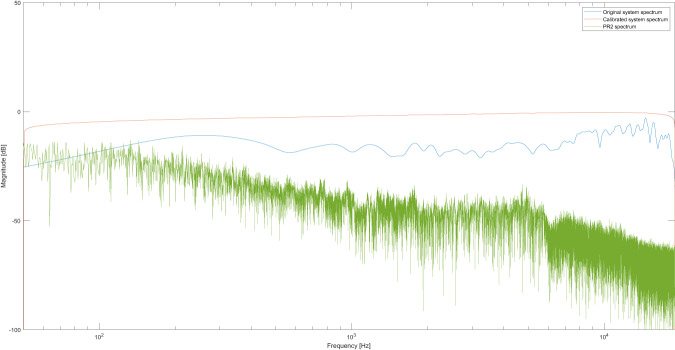
Original, calibrated and PR2 spectrums. The blue line represents the original system spectrum recorded by the electroacoustic system, the orange corresponds to the calibrated spectrum, and the green signal represents the PR2 spectrum, obtained of the measurement at the archaeological site.

To carry out the technical validation of the acoustic measurements, a previous calibration process of the electroacoustic chain used during the measurements was necessary. This calibration prevented that the individual responses of the connected equipment could have affected the acoustic measurements. In addition, the inverse filtering method^39^ was used to adjust the levels and generate a response system as flat as possible. This procedure guaranteed that any acoustic change recorded during the measurements had corresponded exclusively to the response of the system being evaluated, in this case, the archaeological zone of Edzna. The calibration was carried out using a program developed in Matlab, and the implementation code can be consulted in Ibarra *et al*.^39^. The Fig. 7 shows the original system spectrum, the calibrated spectrum, and the PR2 spectrum obtained from the measurement of the point PR2 in the archaeological zone, as a case in point.

## Usage Notes

To read and analyze this data, it is recommended to use Matlab software. Additionally, acoustic parameters can be obtained from this data using the Aurora tool for Audacity, which has been used in previous works^6,8,17,21,22^.

## Data Availability

The authors declare no custom code has been used.

## References

[1] Navas-Reascos G, Alonso-Valerdi LM, Ibarra-Zarate DI (2023). Archaeoacoustics around the World: A Literature Review (2016–2022). Appl. Sci..

[2] Till R (2017). An archaeoacoustic study of the Ħal Saflieni Hypogeum on Malta. Antiquity.

[3] D’Orazio D, Fratoni G, Garai M (2017). Acoustics of a chamber music hall inside a former church by means of sound energy distribution. Can. Acoust. - Acoust. Can..

[4] Martellotta F, Álvarez-Morales L, Girón S, Zamarreño T (2018). An investigation of multi-rate sound decay under strongly non-diffuse conditions: The crypt of the Cathedral of Cadiz. J. Sound Vib..

[5] Katz BFG, Weber A (2020). An Acoustic Survey of the Cathédrale Notre-Dame de Paris before and after the Fire of 2019. Acoustics.

[6] Tronchin L, Merli F, Manfren M (2021). On the acoustics of the Teatro 1763 in Bologna. Appl. Acoust..

[7] Ciaburro G, Berardi U, Iannace G, Trematerra A, Puyana-Romero V (2021). The acoustics of ancient catacombs in Southern Italy. Build. Acoust..

[8] Tronchin L, Merli F, Manfren M, Nastasi B (2020). The sound diffusion in Italian Opera Houses: Some examples. Build. Acoust..

[9] Djordjevic Z, Penezic K, Dimitrijevic S (2017). Acoustic vessels as an expression of medieval music tradition in Serbian sacred architecture. Muzikologija.

[10] D’orazio D, Nannini S (2019). Towards Italian Opera Houses: A Review of Acoustic Design in Pre-Sabine Scholars. Acoustics.

[11] Debertolis P, Gullà D, Piovesana F (2016). Archaeoacoustic research in the ancient castle of Gropparello in Italy. Proc. 5th Virtual Int. Conf. Adv. Res. Sci. Areas.

[12] Debertolis P, Gulla D (2017). Archaeoacoustic Exploration in Montebello Castle (Rimini, Italy). Arts Humanit. Open Access J..

[13] Debertolis P, Ear N, Zivic M (2016). Archaeoacoustic Analysis of Tarxien Temples in Malta. J. Anthropol. Archaeol..

[14] Gerstel SEJ, Kyriakakis C, Raptis KT, Antonopoulos S, Donahue J (2018). Soundscapes of Byzantium: The Acheiropoietos basilica and the Cathedral of Hagia Sophia in Thessaloniki. Hesperia.

[15] Till R (2019). Sound Archaeology: A Study of the Acoustics of Three World Heritage Sites, Spanish Prehistoric Painted Caves, Stonehenge, and Paphos Theatre. Acoustics.

[16] Cox TJ, Fazenda BM, Greaney SE (2020). Using scale modelling to assess the prehistoric acoustics of Stonehenge. J. Archaeol. Sci..

[17] Astolfi, A., Bo, E., Aletta, F. & Shtrepi, L. Measurements of acoustical parameters in the ancient open-air theatre of tyndaris (Sicily, Italy). *Appl. Sci*. **10**, (2020).

[18] Girón S, Galindo M, Romero-Odero JA, Alayón J, Nieves FJ (2021). Acoustic ambience of two roman theatres in the Cartaginensis province of Hispania. Build. Environ..

[19] Galindo M, Girón S, Cebrián R (2020). Acoustics of performance buildings in Hispania: The Roman theatre and amphitheatre of Segobriga, Spain. Appl. Acoust..

[20] Bevilacqua A, Tronchin L (2021). Evaluation of Acoustic Features after Refurbishment Works Inside Two Historical Opera Theatres Located in Italy. Acoustics.

[21] Almagro-Pastor JA, García-Quesada R, Vida-Manzano J, Martínez-Irureta FJ, Ramos-Ridao ÁF (2022). The Acoustics of the Palace of Charles V as a Cultural Heritage Concert Hall. Acoustics.

[22] Umbarkar AS, Nandanwar DV, Chimankar OP (2022). Preliminary Archaeoacoustic Study of Kanheri Caves in Mumbai (Maharashtra, India). Sound Vib..

[23] Kanev N (2020). Resonant Vessels in Russian Churches and Their Study in a Concert Hall. Acoustics.

[24] Sert FY, Karaman ÖY (2021). An Investigation on the Effects of Architectural Features on Acoustical Environment of Historical Mosques. Acoustics.

[25] Debertolis, P. & Gullà, D. Preliminary Archaeoacoustic Analysis of a Temple in the Ancient Site of Sogmatar in South-East Turkey. *Archaeoacoustics Archaeol*. *Sound* **137**, (2016).

[26] Kolar MA, Covey RA, Cruzado Coronel JL (2018). The Huánuco Pampa acoustical field survey: an efficient, comparative archaeoacoustical method for studying sonic communication dynamics. Herit. Sci..

[27] Boren, B., Caro, G., Calixto, D. & González, J. Mexico City’s cathedral: An archaeoacoustical and musicological analysis. *Proc. 22nd Int. Congr. Acoust*. (2016).

[28] Sheets P, Mahoney R (2022). The Soundscape in the Replica of the Cerén Temazcal. Anc. Mesoamerica.

[29] Elkhateeb A, Eldakdoky S (2021). The acoustics of Mamluk masjids: A case study of Iwan-type masjids in Cairo. Appl. Acoust..

[30] Benavides Castillo, A. *Edzná: A Pre-Columbian City in Campeche*. (Instituto Nacional de Antropologia e Historia, 1997).

[31] Zalaquett Rock, F. *Estrategia, comunicación y poder. Una perspectiva social del Grupo Norte de Palenque*. (Universidad Nacional Autónoma de México, 2015).

[32] Declercq NF, Degrieck J, Briers R, Leroy O (2004). A theoretical study of special acoustic effects caused by the staircase of the El Castillo pyramid at the Maya ruins of Chichen-Itza in Mexico. J. Acoust. Soc. Am..

[33] Lubman D (1998). Archaeological acoustic study of chirped echo from the Mayan pyramid at Chichén Itzá. J. Acoust. Soc. Am..

[34] Ramos-Amezquita A, Ibarra-Zarate DI (2013). Acoustic characterization of three archeological sites in the state of Guanajuato, Mexico. Proc. Meet. Acoust..

[35] Blin, G. M. La Convención para la Salvaguardia del Patrimonio Cultural Inmaterial. 157–176 (2006).

[36] Pallán Gayol, C. Secuencia dinástica, glifos-emblema y topónimos en las inscripciones jeroglíficas de Edzná, Campeche (600–900 d.C.): Implicaciones históricas. *Facultad de Filosofía y Letras* (2009).

[37] International Organization for Standardization. ISO 3382-1:2009 Measurement of room acoustic parameters — Part 1: Performance spaces. (2009).

[38] Navas-Reascos G, Wilhelm-deAlba G, Alonso-Valerdi LM, Ibarra-Zarate DI (2023). Mendeley Data.

[39] Ibarra, D., Ledesma, R. & Lopez, E. Design and construction of an omnidirectional sound source with inverse filtering approach for optimization. *HardwareX* **4** (2018).

